# Extensive Extramammary Paget’s Disease With Underlying Perianal Adenocarcinoma: The Role of Neoadjuvant Radiation Treatment

**DOI:** 10.7759/cureus.11966

**Published:** 2020-12-08

**Authors:** Mai N Tran, Jennifer A Harvey

**Affiliations:** 1 Radiation Oncology, Princess Alexandra Hospital Raymond Terrace, Brisbane, AUS; 2 Radiation Oncology, Princess Alexandra Hospital, Brisbane, AUS; 3 Medicine, The University of Queensland, Brisbane, AUS

**Keywords:** extramammary paget’s disease, perianal disease, adenocarcinoma, neoadjuvant radiation treatment

## Abstract

Extramammary Paget’s disease (EMPD) is an uncommon entity, and secondary EMPD is even rarer. To our knowledge, this report involves one of the only three cases in the literature to date regarding the use of neoadjuvant radiation therapy in the treatment of secondary EMPD. A 65-year-old woman’s EMPD had become more widespread over the years to involve buttocks, perineum, anus, vulva, and vagina. Given the knowledge of potential secondary EMPD, suspicious perianal lesions were biopsied. Histology and immunohistochemistry staining confirmed adenocarcinoma. Our patient was treated with neoadjuvant radiation therapy, along with concurrent chemotherapy. This was followed by pelvic exenteration, which confirmed a complete response from the neoadjuvant treatment. We discuss her presentation, investigations, and treatment regimen in detail. In addition, we review the treatment of secondary EMPD as reported in previously published literature.

## Introduction

Extramammary Paget’s disease (EMPD) is an uncommon entity, and only scattered cases have been reported in the literature so far. The concept of primary and secondary EMPD disease treatment has been established in such works. This case report highlights the importance of keeping secondary EMPD in mind in cases where the condition does not improve with conservative management. In addition, we also emphasise the need for close monitoring in such cases to ensure that any potential malignancy is not missed.

## Case presentation

A 65-year-old woman with extensive EMPD of the buttocks was initially managed by her general practitioner and then by a dermatology clinic. Her management included surveillance, repeated biopsies, topical imiquimod, topical fluorouracil, photodynamic therapy, laser treatment, and debulking. She was largely asymptomatic, aside from occasional itching. Her past medical history included chronic hepatitis B, for which she was on entecavir.

**Figure 1:**
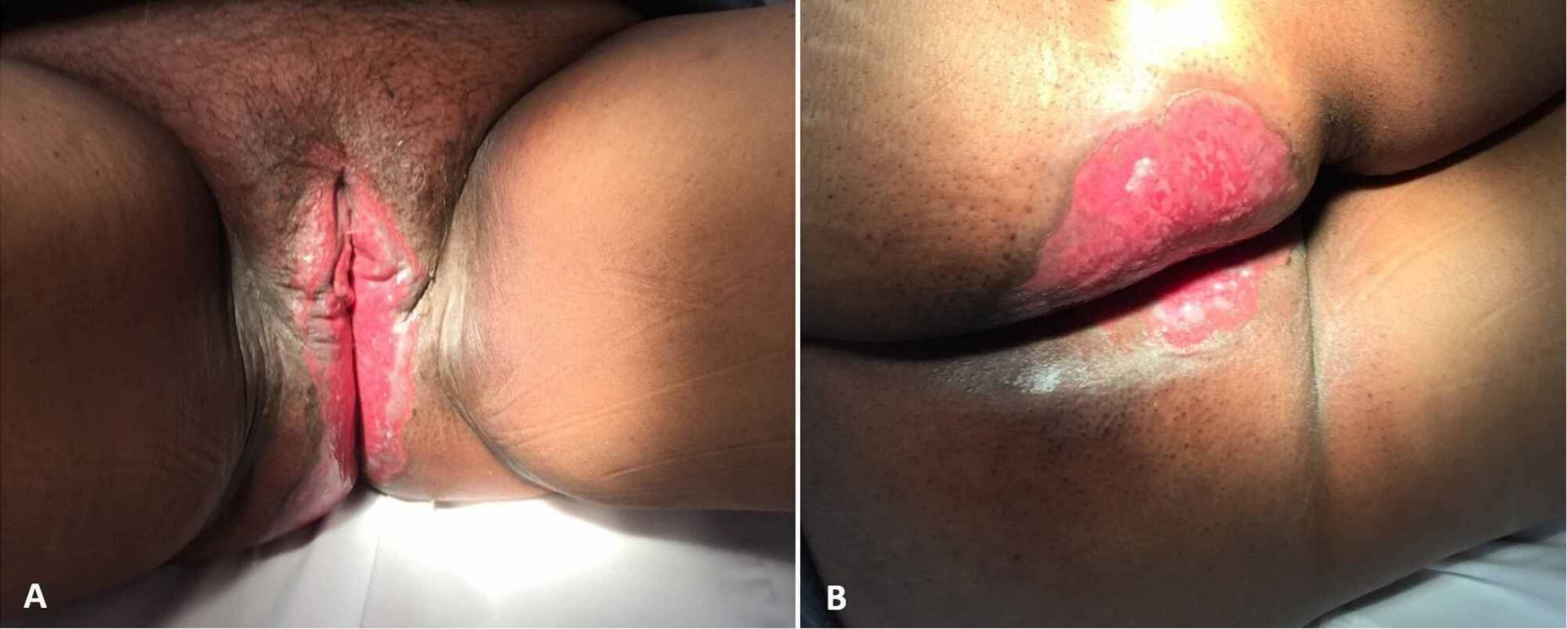
Ulcerated appearance of Paget’s disease overlying a significant surface area at the time of referral to radiation oncology A) affecting the vulva and the perineum, B) affecting the perianal region and right buttock

**Figure 2:**
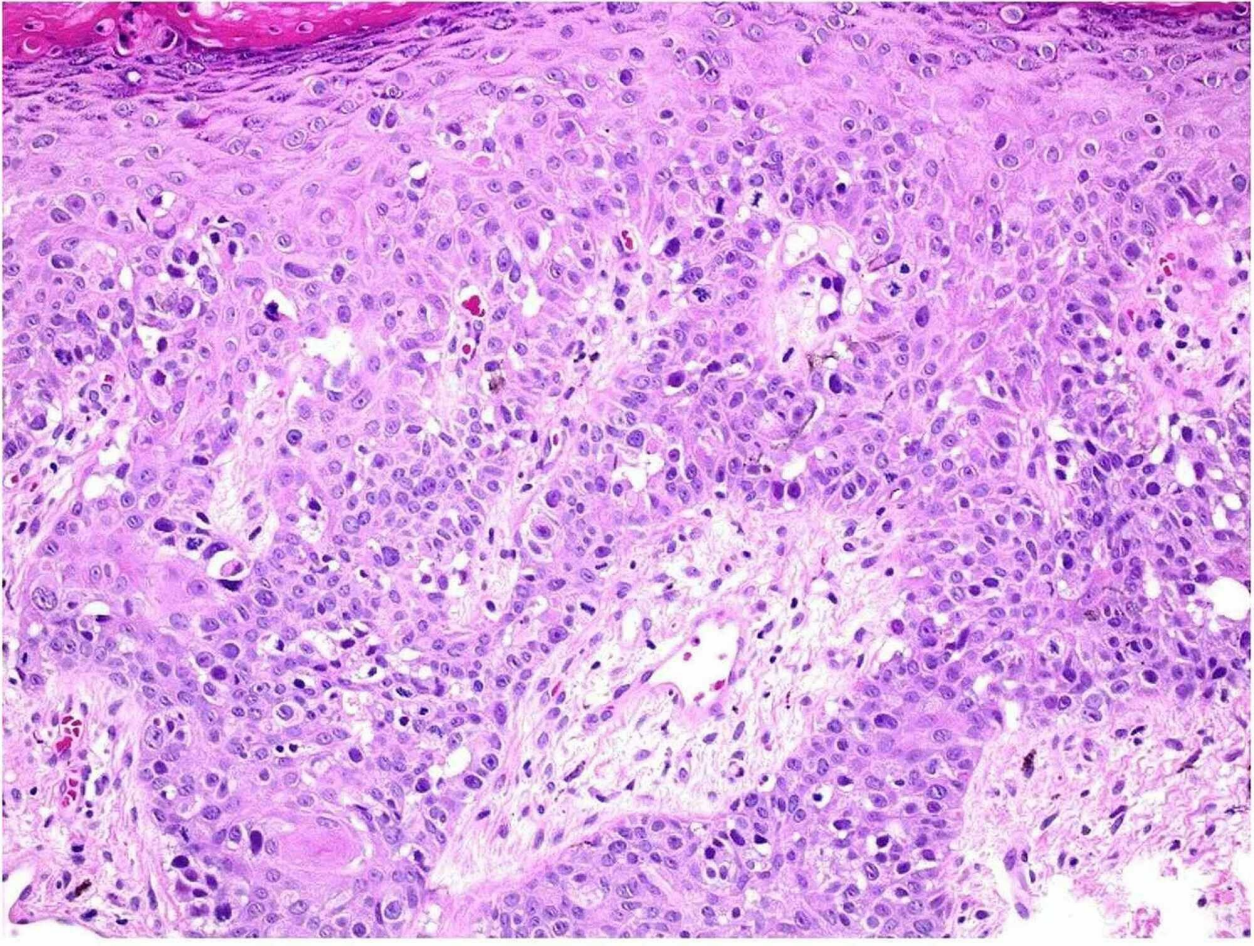
Infiltration of squamous epithelium with single atypical cells with frequent mitotic figures and PAS-positive intracytoplasmic vacuoles The atypical cells were positive for CK7, CK20, CDX2, EMA, and CEA on immunohistochemistry. These findings are diagnostic of Paget’s disease, most likely from a rectal adenocarcinoma PAS: periodic acid-Schiff

After six years of conservative treatment, her disease had progressed to become more symptomatic and more widespread, extending to her buttocks bilaterally, perineum, anus, vulva, and vagina (Figure 1). She went on to have an examination under anaesthetic, mapping biopsies of the vulva, vagina, and perianal area, as well as colposcopy and colonoscopy. Her disease was visualised in the left anterior vagina, bilateral labia, and perianal regions. Her skin was badly indurated and was rubbery to touch. There were large areas of ulceration, particularly on the right buttock, which measured 10 cm. There were two highly suspicious lesions on mapping biopsies at 11 and 12 o’clock in the perianal area. The histopathology from the biopsies from these sites confirmed adenocarcinoma (Figure 2). There was no mismatch repair gene deficiency detected, and human epidermal growth factor receptor 2 (HER2) was negative. The biopsies in the other areas of involvement were characteristic of Paget’s disease, with signet ring morphology and intraepithelial atypical cells with cytoplasmic mucin. Paget’s disease was confirmed via immunohistochemistry with positive Cam 5.2, CK7, CK20, CDX2, EMA, and CEA staining.

**Figure 3:**
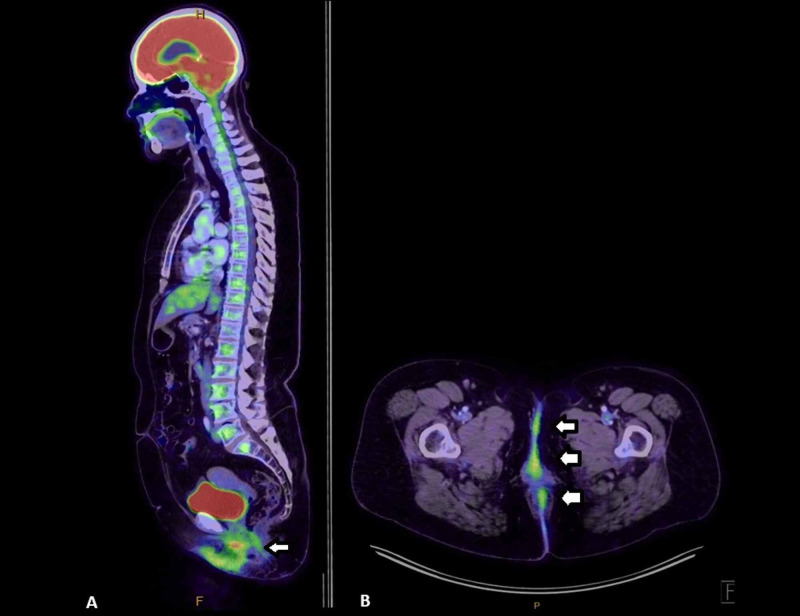
Varying degrees of increased FDG activity around the perianal region, natal cleft, and anterior perineum (white arrows) on PET imaging A) sagittal plane. B) axial plane PET: positron emission tomography

A positron emission tomography (PET) scan and MRI of the pelvis revealed mild perianal skin and subcutaneous thickening with varying degrees of FDG activity, changes consistent with cutaneous perianal Paget’s (Figure 3). There was no mass-like component identified on the MRI. However, there was mild FDG avidity in the midline, with an avid right inguinal node. A fine needle aspirate of this node showed no malignant cells on cytology.

Discussion at the colorectal multidisciplinary team (MDT) meeting at our hospital about the management of adenocarcinoma in the perianal region within the field of EMPD resulted in a recommendation for a long-course chemoradiation. This would then be followed by restaging prior to a decision regarding surgery. It was initially hoped that definitive chemoradiation could avoid the need for very extensive surgery. As this is a rare disease, the patient’s further treatment was discussed in a second MDT meeting specialising in pelvic exenterative surgery. The decision to proceed to pelvic exenteration was driven by the large volume of Paget’s disease that was already involved and the underlying malignancy in the perianal area, conferring a worse prognosis for local recurrence. It would also have been much more difficult to perform salvage surgery for recurrence in the future due to late radiation changes.

**Figure 4:**
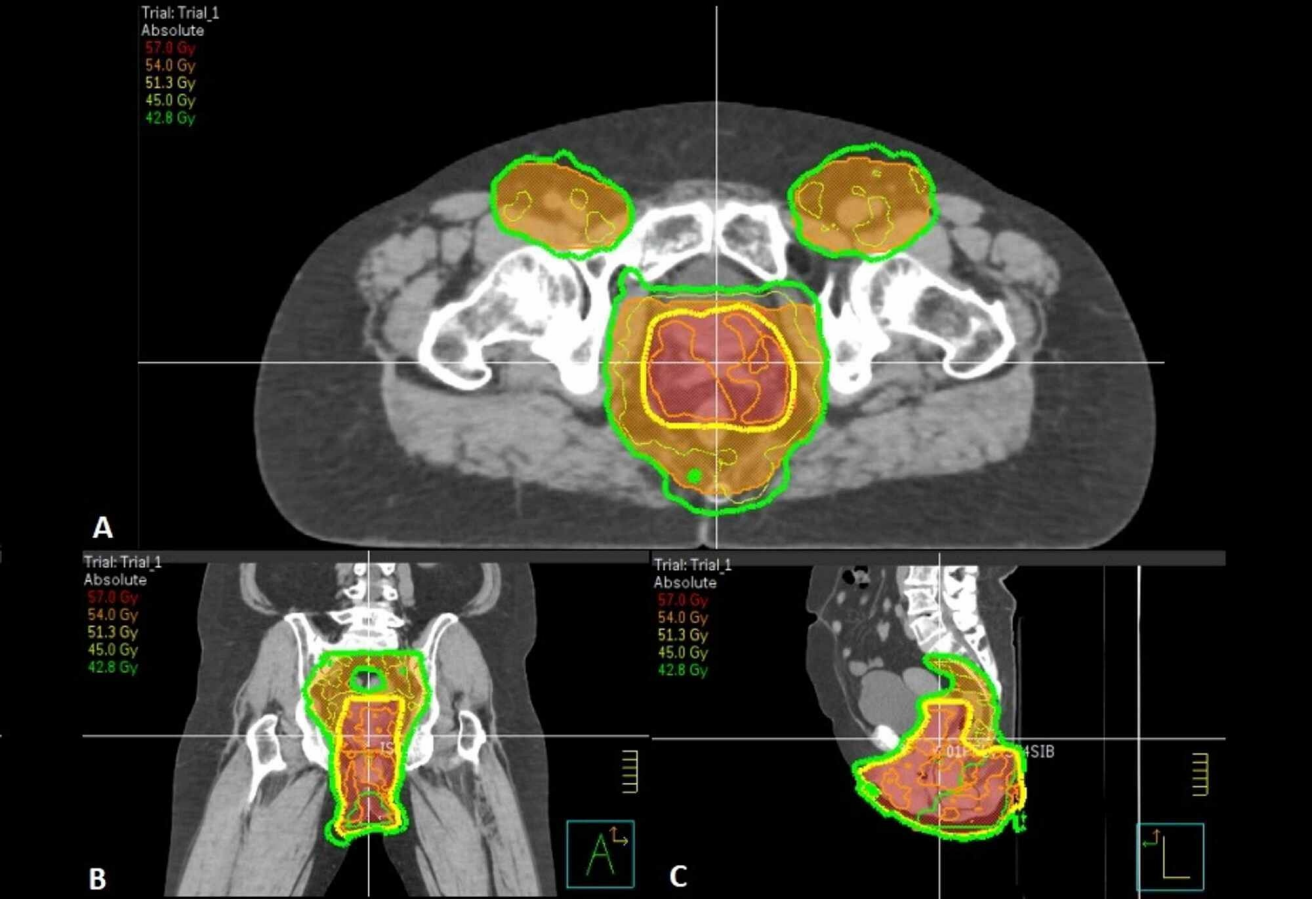
Dosimetric plan showing the various doses within the pelvis in Gy, with the corresponding colour legend located on the top-left hand corner CT planning scan in A) axial plane, B) coronal plane, and C) sagittal plane CT: computed tomography

Given that there was a component of invasive disease in the perianal area, the treatment took the form of six weeks of concurrent chemoradiation. The radiation volumes included all areas involved with Paget’s disease and invasive carcinoma, with the vulva, vagina, perianal region, anal canal, and buttocks receiving 54 Gy in 30 fractions (Figure 4). Bolus (tissue-equivalent material) was used over involved areas to bring the dose onto the skin. The uninvolved rectum and regional nodes (including mesorectal, presacral internal, external iliac, and inguinal nodes) were treated to 45 Gy in 25 fractions. A 1.5-2 cm margin was used around the gross tumour, avoiding dose to the patient's medial thighs superiorly. The radiation therapy was delivered via the volumetric modulated arc therapy (VMAT) technique for conformality and to spare the normal tissues. The concurrent chemotherapy took the form of oral capecitabine, in which 1,650 mg was taken twice a day (825 mg/m^2^ bd) for five days per week.

The patient was seen on a weekly basis initially, escalating to twice weekly towards the end of her chemoradiation. She was continued to be seen weekly for four weeks until her skin reaction had healed. On day one of radiation therapy, she began the use of emollient cream to her buttocks and perianal area. After 12 out of 30 fractions, the patient began to experience dysuria, which was managed with a urinary alkaliniser. After 23 fractions, the breakdown of the perianal and vulval skin required additional management with mixed lignocaine and hydrogel. After the completion of radiation therapy, there was moist desquamation (grade 3 skin reaction) involving the bolused areas. This was managed with zinc and castor oil cream covered with a non-stick dressing, combine pads, and pull-up disposable underpants. The patient was advised to have salt baths, regular paracetamol, and oxycodone as required.

After six weeks, most of her buttock skin was healed, but there was still a grade 3 reaction with residual moist desquamation in the perianal and vulvar regions. After three months, the radiation-induced skin reaction had completely healed.

Approximately five months after the completion of neoadjuvant therapy, allowing for healing and hospital waiting lists, the patient proceeded to have a pelvic exenteration: open abdominoperineal resection with en block total cystectomy, urethrectomy, hysterectomy, bilateral salgingo-oophorectomy, total vaginectomy, colonic urinary conduit, and vertical rectus abdominis myocutaneous (VRAM) flap. This histopathology identified no residual tumour (ypT0 ypN0), with 0/13 lymph nodes involved.

The patient was admitted to ICU for four days post-surgery for haemodynamic support and pain management infusions (lignocaine and ketamine). Her total hospital admission spanned six weeks, with the last week of this time spent in the rehabilitation unit. Ten days after her surgery, the patient required a further laparotomy and stoma revision for a necrotic end colostomy. The medical oncologists advised that there was no role for adjuvant chemotherapy. The patient was doing well during her last review (14 months after the operation). She was asymptomatic and managing her stomas well. At her latest review, there was some evidence of fibrotic and hyperpigmentation changes at the periphery of the flap reconstruction on her buttocks. We plan to follow her up every six months with clinical examination and MRI of the pelvis annually, to monitor her for late radiation side effects and recurrence, with a total planned follow-up duration of five years.

## Discussion

EMPD can be subdivided into either primary or secondary forms. Primary EMPD is a primary cutaneous condition confined to the epidermis. Secondary EMPD constitutes cutaneous involvement via extension from an underlying carcinoma, most likely of lower gastrointestinal, vulvar, or urinary tract origin.

EMPD mostly affects Caucasian females in their 60s-70s [1]. The presentation mainly involves pruritus; other symptoms include pain and bleeding [2]. On examination, EMPD appears similar to eczema, with slightly raised and well-demarcated edges on a red background. The overlying surface appears crusted, leukoplakia-like, or erosive. There are often pale islands dotted throughout. Common areas affected by EMPD include the vulva, perineum, perianal, scrotum, and penile skin (in descending frequency). Perianal lesions are considered the most aggressive form [2].

Given EMPD appears as an inflammatory skin disease, its differentials include eczema, dermatitis (contact or seborrheic), and superficial fungal infections [3]. It is pertinent to pursue a diagnosis for suspicious lesions that persist or fail to respond to therapy for such conditions. Dermoscopy may be used as the initial tool to differentiate EMPD from benign inflammatory skin disease, given the differing vascular patterns of EMPD.

EMPD is a histopathologic diagnosis. Paget’s cells consist of large nuclei, with a prominent vesicular nucleus within an abundant pale cytoplasm [4]. Immunohistopathology stains are positive for CAM 5.2 and carcinoembryonic antigen [3]. Given the abundant mucin in the cytoplasm of Paget’s cells, staining is positive for periodic acid-Schiff (PAS) and Alcian blue [3]. Generally, primary EMPD stains are positive for CK7 and gross cystic disease fluid protein-15 (GCDFP-15) and negative for CK20 [3]. Secondary EMPD stains are positive for CK20 and negative for CK7 and GCDFP-15. Of note, some cases have been incongruent with these generalisations, and therefore the above should be considered as generalisations. Signet ring cells are seen in secondary EMPD associated with rectal adenocarcinoma [5]. Positive stains suggesting gastrointestinal cancer include CK20 and CDX2 [6,7]. Some cases exhibit HER2 overexpression, and hence a potential therapeutic target [8].

Treatment of primary EMPD consists of surgical excision as the standard of treatment, with a preferred 2 cm margin [4]. Mohs micrographic surgery (MMS) in particular is a surgical technique associated with lower recurrence rates [9]. However, it has been observed that MMS can be challenging and time-consuming when EMPD covers large areas. Given the invasive nature of secondary EMPD, aggressive surgery such as abdominoperineal resection has been recommended [10]. Local recurrence varies widely in the order of 15-61% [4]. With MMS, the local recurrence rate is 8-28% [4]. These rates may be due to the multicentric nature of EMPD and/or microscopic disease extension beyond clinically visible borders.

Radiation therapy can be used as an alternative to surgery in inoperable cases [3]. The goal of radiation therapy is to achieve tumour control. Burrows et al. [11] and Moreno-Arias et al. [12] have reported no disease recurrence during a follow-up of three to four years after definitive radiation in their small cohorts of patients with EMPD, without underlying carcinoma. Besa et al. [13] have reported the same success for such patients, except for local recurrence in one of two of their patients with underlying carcinoma post-definitive radiation therapy. Doses that have been reported in the literature are mainly 60 Gy to the gross tumour volume, with a 2 cm margin [14]. Of note, there have been no randomised controlled trials directly comparing surgical excision with definitive radiotherapy for the treatment of EMPD. It has been suggested that those patients with dermal invasion undergo prophylactic lymph node irradiation, given regional lymph node metastasis occurs in higher frequency in those with dermal invasion. In our patient, we chose a dose of 54 Gy in 30 treatments with a lower prophylactic dose of 45 Gy to nodal regions that would not have been addressed surgically. Treating such a large volume required a balance between local control and toxicity.

Adjuvant radiation therapy has also been recommended, especially in cases of multifocal disease, lymph node metastasis, positive surgical margins, and associated malignancies. Hata et al. [15] have reported the outcome of 21 patients with EMPD treated with postoperative radiation therapy (PORT), with a dose range of 45-64.8 Gy in 23-36 fractions. Six out of 21 patients developed distant metastasis during the median follow-up period of 38 months. It was highlighted that these six patients had dermal invasion and involved lymph nodes prior to PORT. Hata et al. [16], in a separate study, have published the outcome of eight patients with EMPD, with lymph node metastasis treated with PORT. The total dose ranged from 45 to 61.2 Gy in 25-34 fractions. Half of these patients were found to have experienced recurrence at a follow-up at 22 months, one with the progression of irradiated lymph node (treated to 45 Gy) and three with distant metastasis. Another publication by Hata et al. [17] has reported the use of PORT in eight patients with positive surgical margins. The total dose ranged from 45 to 70.2 Gy in 25-39 fractions. Five of these patients developed recurrence outside the radiation field. Tagliaferri et al. [18] performed a systematic review in 2018, which included 19 articles published between 1986 and 2017. These publications were retrospective analyses only, and there were no randomised controlled trials. These studies included a spectrum of different radiation doses, fractionation, techniques, and clinical scenarios. Of note, it was found that the complete response rate ranged from 50 to 100% for radiation therapy after surgery, for the treatment of primary or recurrent disease.

In our case, radiation therapy was used in the neoadjuvant setting prior to surgery. Given the presence of underlying adenocarcinoma and extensive surface area of disease, it was felt that there was a high risk of local recurrence with single-modality treatment alone. To our knowledge, this is the third case in the literature about neoadjuvant radiation therapy in the treatment of secondary EMPD. Tagliaferri et al. [18], in a systematic review in 2018, identified two other publications reporting single cases using neoadjuvant radiation therapy. In 2013, Cai et al. [19] reported a gynaecological series, with a single case of a patient with vulval cancer and confirmed extensive retroperitoneal lymph node metastases. This patient was treated with external beam radiation to a dose of 43.30 Gy followed by two cycles of chemotherapy and then surgery. No toxicity or outcome data were included. Besa et al. [13] in 1992 reported on a patient treated with external beam radiation to a dose of 40 Gy/20 fractions over four weeks. The patient was treated with opposed anterior and posterior fields. The patient suffered necrosis in the perineal wound after an abdominal perineal resection. Local control was obtained, but the patient died of disseminated rectal cancer.

There is an acknowledgment from the literature that secondary EMPD is rare and knowledge about the use of neoadjuvant radiation therapy in this setting is scarce. In our case, we used higher doses of radiation in comparison to these two papers. Advanced techniques of radiation planning and conformal radiation delivery with VMAT or intensity-modulated arc therapy (IMRT) has allowed dose escalation while sparing normal tissues. Our treatment regimen resulted in acceptable toxicities. There was never a break in radiation treatment at any point, and hospitalisation was not required. As outlined earlier, our patient healed well, and local control has been maintained to date.

Radiation therapy in the palliative setting for EMPD is often for symptom control in the areas of metastatic spread, such as the spine. Moretto et al. [20] have reported the utilisation of a common palliative regime of 20 Gy in five fractions in such a setting.

The use of concurrent chemotherapy has also been described [2]. However, there is no established standard regimen. In addition, targeted therapy can be considered where appropriate. Of note, 20-60% of EMPD cases display HER2 protein overexpression [2]. This would take the form of a recombinant monoclonal antibody against HER2 such as trastuzumab.

Poor prognostic markers include lymph node metastasis, dermal invasion, tumour thickness, advanced stage, positive surgical margins, lymphovascular involvement, and high Ki-67 expression [4]. Lymph node metastasis and dermal invasion have been reported to have significant impacts on distant metastasis-free rates and overall survival [14]. At three years, distant metastasis-free rates are 33% and 89% in patients with and without regional lymph node metastasis respectively, and for dermal invasion, 51% and 92% for with and without respectively [14]. At five years, overall survival rates are 18% and 93% in patients with and without regional lymph node metastasis, and for dermal invasion, 47% and 100% for with and without respectively [14].

Long-term follow-up is integral in the management of EMPD, as up to 25% of patients may have an associated underlying carcinoma [4]. Regular inspection with a low threshold for a biopsy is recommended. Case discussion in an MDT meeting is also integral in the management of patients with EMPD. A clear management plan can be created with the approval of a number of different subspecialties through such discussions.

## Conclusions

In conclusion, secondary EMPD is an uncommon condition with a link to underlying malignancy. It is derived from direct invasion from visceral organ carcinoma to the dermis. Therefore, when the anus, urethra, or vagina are involved, an underlying malignancy of these visceral organs should be kept in mind. This is of particular importance for diseases involving the perianal region, given the significantly low overall survival associated with this area. The preinvasive phase may be prolonged (two to eight years), with 40% of lesions progressing to invasive cancer.

This case report is the third one in the literature to detail the use of radiation therapy in the neoadjuvant setting in secondary EMPD from perianal adenocarcinoma. In our case, we demonstrated the acceptability of using higher doses of radiation therapy than previously documented. These doses resulted in local control to date, with no impedance to wound healing, and yielded acceptable both acute and late toxicity. In the future, a dose of 50 Gy in 25 fractions to the gross disease and 45 Gy in 25 fractions to prophylactic volumes would be less toxic and result in shorter recovery and would be recommended as a preoperative dose if surgery was planned from the beginning.

## References

[1] Parker LP, Parker JR, Bodurka-Bevers D, Deavers M, Bevers MW, Shen-Gunther J, Gershenson DM (2000). Paget's disease of the vulva: pathology, pattern of involvement, and prognosis. Gynecol Oncol.

[2] Sisodia S, Boushey R, Lee G, Marginean C, Gomes MM, Bhattacharya G, Dennis K (2017). Perianal pagetoid intraepithelial carcinoma. Case Rep Gastroenterol.

[3] Ito T, Kaku-Ito Y, Furue M (2018). The diagnosis and management of extramammary Paget's disease. Expert Rev Anticancer Ther.

[4] Shepherd V, Davidson EJ, Davies-Humphreys J (2005). Extramammary Paget’s disease. BJOG.

[5] Goldblum JR, Hart WR (1998). Perianal Paget's disease: a histologic and immunohistochemical study of 11 cases with and without associated rectal adenocarcinoma. Am J Surg Pathol.

[6] Ohnishi T, Watanabe S (2000). The use of cytokeratins 7 and 20 in the diagnosis of primary and secondary extramammary Paget's disease. Br J Dermatol.

[7] De Nisi MC, D'Amuri A, Toscano M, Lalinga AV, Pirtoli L, Miracco C (2005). Usefulness of CDX2 in the diagnosis of extramammary Paget disease associated with malignancies of intestinal type. Br J Dermatol.

[8] Hatta N, Yamada M, Hirano T, Fujimoto A, Morita R (2008). Extramammary Paget's disease: treatment, prognostic factors and outcome in 76 patients. Br J Dermatol.

[9] O'Connor WJ, Lim KK, Zalla MJ, Gagnot M, Otley CC, Nguyen TH, Roenigk RK (2003). Comparison of mohs micrographic surgery and wide excision for extramammary Paget's disease. Dermatol Surg.

[10] Lian P, Gu WL, Zhang Z (2010). Retrospective analysis of perianal Paget's disease with underlying anorectal carcinoma. World J Gastroenterol.

[11] Burrows NP, Jones DH, Hudson PM, Pye RJ (1995). Treatment of extramammary Paget's disease by radiotherapy. Br J Dermatol.

[12] Moreno-Arias GA, Conill C, Castells-Mas A, Arenas M, Grimalt R (2001). Radiotherapy for genital extramammary Paget's disease in situ. Dermatol Surg.

[13] Besa P, Rich TA, Delclos L, Edwards CL, Ota DM, Wharton JT (1992). Extramammary Paget's disease of the perineal skin: role of radiotherapy. Int J Radiat Oncol Biol Phys.

[14] Hata M, Koike I, Wada H (2014). Radiation therapy for extramammary Paget disease: treatment outcomes and prognostic factors. Ann Oncol.

[15] Hata M, Koike I, Wada H (2015). Postoperative radiation therapy for extramammary Paget's disease. Br J Dermatol.

[16] Hata M, Koike I, Wada H (2014). Radiation therapy for lymph node metastasis from extramammary Paget's disease. J Eur Acad Dermatol Venereol.

[17] Hata M, Omura M, Koike I (2011). Role of radiotherapy as curative treatment of extramammary Paget's disease. Int J Radiat Oncol Biol Phys.

[18] Tagliaferri L, Casà C, Macchia G (2018). The role of radiotherapy in extramammary Paget disease: a systematic review. Int J Gynecol Cancer.

[19] Cai Y, Sheng W, Xiang L, Wu X, Yang H (2013). Primary extramammary Paget's disease of the vulva: the clinicopathological features and treatment outcomes in a series of 43 patients. Gynecol Oncol.

[20] Moretto P, Nair VJ, Hallani SE, Malone S, Belanger E, Morash C, Canil CM (2013). Management of penoscrotal extramammary Paget disease: case series and review of the literature. Curr Oncol.

